# Identification of Prognostic Biomarkers for Multiple Solid Tumors Using a Human Villi Development Model

**DOI:** 10.3389/fcell.2020.00492

**Published:** 2020-06-23

**Authors:** Botao Zhang, Yuanjing Wang, Hongxia Li, Lin Feng, Wenbin Li, Shujun Cheng

**Affiliations:** ^1^Department of Neuro-oncology, Neurosurgery Center, Beijing Tiantan Hospital, Capital Medical University, Beijing, China; ^2^State Key Laboratory of Molecular Oncology, Department of Etiology and Carcinogenesis, National Cancer Center/National Clinical Research Center for Cancer/Cancer Hospital, Chinese Academy of Medical Sciences and Peking Union Medical College, Beijing, China; ^3^Department of Gynecological Oncology, Beijing Obstetrics and Gynecology Hospital, Capital Medical University, Beijing, China; ^4^Department of Obstetrics and Gynecology, Beijing Shijitan Hospital, Capital Medical University, Beijing, China

**Keywords:** villi, solid tumor, gene co-expression networks, off-track, prognosis

## Abstract

The processes of embryonic development that rely on epithelial-mesenchymal transition (EMT) for the implantation of trophoblast cells are co-opted by tumors, reflecting their inherent uncontrolled characteristics and leading to invasion and metastasis. Although tumorigenesis and embryogenesis have similar EMT characteristics, trophoblasts have been shown to exhibit “physiological metastasis” or be “pseudo-malignant,” resulting in different outcomes. The gene co-expression network is the basis of embryonic development and tumorigenesis. We hypothesize that if the gene co-expression network in tumors is “off-track” from that in villi, it is more likely to develop into malignant tumors and have a worse prognosis, and we proposed the “off-track theory” for the first time. In this study, we examined gene co-expression networks in villi and multiple solid tumors. Through network functional enrichment analyses, we found that most tumors and villi exhibited a significantly enriched EMT, but the genes that performed this function were not identical. Then, we identified the “off-track genes” in the EMT-related gene interaction network using the “off-track theory,” and through survival analysis, we discovered that the risk score of “off-track genes” was associated with poor survival of cancer patients. Our study indicated that villi development is a reliable and strictly regulated model that can illuminate the trajectory of human cancer development and that the gene co-expression networks in tumor development are “off-track” from those in villi. These “off-track genes” may have a substantial impact on tumor development and could reveal novel prognostic biomarkers.

## Introduction

Cancer is a complex disease with various pathologic properties (Machnik et al., 2019), and cancer incidence and mortality have been increasing, making cancer the leading cause of death and a major public health problem worldwide (Chen et al., 2016; Siegel et al., 2018). Although multiple forms of treatment exist for cancers, such as surgery, chemotherapy, radiotherapy, targeted therapy, and so on, the prognosis of patients with cancer is still poor, and cancer still poses a serious threat to human health and affects the quality of life of patients (Kovar et al., 2016; Gigic et al., 2018). In addition, there is a strong heterogeneity in tumor tissues, and the genome is extremely unstable (Burrell et al., 2013; Troiani et al., 2014). Therefore, a model similar to the biological behavior and molecular characteristics of tumor cells but without heterogeneity is urgently needed to study tumors.

Tumorigenesis and placental villi development have many similar biological behaviors, especially during the early stage of pregnancy (Strickland and Richards, 1992; Holtan et al., 2009; Perry et al., 2009). The epithelial-mesenchymal transition (EMT) plays an important role in embryonic development and cancer progression (Chaffer et al., 2016; Simeone et al., 2019). The EMT is a fundamental cell biological process that plays a critical role in embryogenesis, which was reactivated during cancer progression and enhances the metastatic phenotype. Implantation of trophoblast cells of the placenta into the decidual and muscular layers of the uterus is the *sine qua non* of a successful pregnancy, and trophoblast cells promote implantation through EMT, which results in loss of cell-to-cell contact inhibition (Holtan et al., 2009). EMT of tumor cells in cancer progression possibly generates the different cell types of the tumor stroma, such as cancer-associated myofibroblasts. EMT contributes to new tumor cell properties required for invasiveness and vascular intravasation during metastasis (Moustakas and Heldin, 2010).

The processes of implantation during placental development that rely on EMT are co-opted by tumors, reflecting their inherent uncontrolled characteristic, which leads to invasion and metastasis (Hay, 2005; Huber et al., 2005; Jean Paul and Sleeman, 2006). However, tumorigenesis and placental development have different outcomes. Placental development is a physiological process; the placenta is an important organ used to nourish the fetus during pregnancy, and the EMT process is strictly regulated. However, tumorigenesis is a pathological process, and the EMT process is not regulated by the host. Disequilibrium is the key feature of cancer occurrence and development (Hanahan and Weinberg, 2011; Macklin et al., 2017). In addition, the gene regulatory network induces the expression of other target genes via the protein products of differentially expressed genes, which is the basis of many biological processes, such as embryonic development and tumorigenesis (Singh et al., 2018). During the process of placental development, a strong correlation exists between different genes, suggesting that all biological functions could be modulated through the precise regulation of gene networks (An et al., 2015). However, during the process of tumor progression, close gene regulation relationships are abolished, and the correlations between genes responsible for normal placental development are disrupted. Based on this principle, we proposed the “off-track theory” for the first time. Such “off-track genes” are likely to promote the progression of cancer and may also predict the prognosis of cancer patients. Therefore, we deemed that in placental villi development and tumorigenesis, the gene regulatory networks are altered, resulting in gene co-expression disorders and different outcomes. In summary, when the gene co-expression network in tumors is “off-track” from that in villi development, cells are more likely to develop into malignant tumors, or have a worse prognosis.

In this study, we utilized RNA sequencing data combined with RNA transcriptome data of multiple solid tumors from The Cancer Genome Atlas (TCGA) to construct gene co-expression networks for chorionic villi and multiple solid tumors, respectively. Then, according to the “off-track theory,” we identified “off-track genes” from the EMT-related gene co-expression networks and then screened “off-track genes” that were highly expressed in cancers. Afterward, we performed a survival analysis to assess whether these “off-track genes” could predict the prognosis of cancer patients.

Our research indicated that placental villi development is a reliable and strictly regulated model. The villi development model could produce new insights for the understanding of tumors and provide new methods for cancer research. Additionally, through the different co-expression relationships between physiological and pathological states, we found new biomarkers which may be helpful for the treatment and post-treatment monitoring of cancers.

## Materials and Methods

### Patients, Tissue Samples, and TCGA RNA Sequence Data Curation

Tissue samples of developing villi were obtained from Beijing Shijitan Hospital between March 2015 and August 2016. The samples included 15 chorionic villus samples at 6 to 10 weeks of gestation (3 samples at each time point) and 6 leaf chorionic samples from postpartum placental tissue representing the mature placenta. The detailed information regarding those samples is presented in Supplementary Table S1. The inclusion criteria for the pregnant women have been described previously (Zhang et al., 2017). All donors signed informed consent forms. This study was reviewed and approved by the Ethics Committee of the National Cancer Center/Cancer Hospital, Chinese Academy of Medical Sciences, and Peking Union Medical College.

For TCGA datasets, RNA sequencing data of bladder urothelial carcinoma (BLCA), breast invasive carcinoma (BRCA), colon adenocarcinoma (COAD), rectum adenocarcinoma (READ), esophageal adenocarcinoma (EAC), lung adenocarcinoma (LUAD), stomach adenocarcinoma (STAD), glioma (GBMLGG), head and neck squamous cell carcinoma (HNSC), esophageal squamous cell carcinoma (ESCC), and lung squamous cell carcinoma (LUSC) and the corresponding clinical information were downloaded through the R package “TCGA2STAT.” Detailed information on these 11 types of tumors is shown in Table 1.

**TABLE 1:** The sample numbers of these 11 types of tumors.

Tumor	Stage I/WHO I	Stage II/WHO II	Stage III/WHO III	Stage IV/WHO IV	NA	Normal
BLCA	2	130	140	134	2	19
BRCA	181	620	250	20	22	112
COAD	44	108	79	39	9	41
EAC	12	23	29	5	20	11
ESCC	7	55	27	4	2	11
GBMLGG	0	515	0	152	0	5
HNSC	27	74	81	266	72	44
LUAD	269	120	80	25	8	59
LUSC	244	162	84	7	4	51
READ	12	26	33	13	9	10
STAD	52	121	165	37	13	35

We also collected datasets from the Gene Expression Omnibus (GEO) and Chinese Glioma Genome Atlas (CGGA) for validation, including 3 independent sets of COAD (GSE14333, GSE12945, and GSE17536), 3 independent sets of LUAD (GSE30219, GSE13213, and GSE68465), 3 independent sets of GBMLGG (GSE109857, GSE16011, and CGGA), and their corresponding clinical information. All of the datasets were downloaded and analyzed directly.

### RNA-Seq and Analysis

Total RNA was isolated from frozen chorionic villus and mature placenta tissues with TRIzol reagent (Thermo Fisher, United States) according to the manufacturer’s instructions. A complementary DNA library was prepared, and sequencing was performed according to the Illumina standard protocol by Beijing Novel Bioinformatics Co., Ltd.^1^ Raw reads from the RNA-seq libraries were trimmed to remove the adaptor sequence, reads with adaptor contaminants, and low-quality reads. The indexes of the reference genome were built using Salmon, and the paired-end clean reads were aligned to the reference genome using UCSC version hg19. The transcripts per million (TPM) value of each gene was calculated based on the gene read counts mapped to that gene. The raw sequence data of villus tissues reported in this paper have been deposited in the Genome Sequence Archive (Genomics, Proteomics & Bioinformatics 2017) in the Beijing Institute of Genomics (BIG) Data Center (Nucleic Acids Res 2018), BIG, Chinese Academy of Sciences, under the accession number HRA000050.

### Construction of Gene Co-expression Networks and Enrichment Analysis

For chorionic villus and multiple cancer datasets, we used the “MatrixEQTL” package in R to compute all pairwise associations of gene expression levels and to estimate false discovery rates (FDRs; Shabalin, 2012). To increase the computational speed, FDRs were only reported for interactions achieving a raw *p*-value less than 0.001, and in subsequent analyses the self-interactions were removed from the co-expression networks.

For chorionic villus and multiple cancer gene co-expression interactions, we selected the 10,000 most significant co-expression relationships to build the networks and perform enrichment analysis. We used the “iGraph” package to compute each node’s degree (Netzer et al., 2011) and the “RedeR” package to visualize the networks (Castro, 2012). Functional enrichment analyses of networks were performed with the Spatial Analysis of Network Associations (SANTA) package using custom gene sets (Cornish and Markowetz, 2014), including 50 hallmark gene sets from the Molecular Signatures Database (MSigDB).

### Identification of “Off-Track Genes”

Villi development and cancer progression exhibit many similar biological behaviors but have distinct outcomes. For the first time, we proposed the “off-track theory” to explore the genes that are out of balance in cancers.

First, genes related to EMT were assessed in villi and tumors. We supposed that Pearson’s correlation coefficient of a gene-pair (gene A, B) in the villi is *x*, and that of the gene-pair in cancer is y. Then, we projected this onto a coordinate axis as a point (*x*, *y*). When the correlation coefficients were equal, the point was projected on the line *y* = *x* in the coordinate axis. When a point (*x*, *y*) deviated from the line *y* = *x*, this indicated that the gene interaction was more “off-track” in cancer progression than in the normal physiological process, resulting in a larger contribution to the synergistic disorder. We defined genes with a distance from the point (*x*, *y*) to the line *y* = *x* greater than 1 as “off-track genes.”

To further identify genes related to the prognosis of cancer patients among “off-track genes,” we used Student’s *t*-test to screen for genes that are highly expressed in cancers.

### Survival Analysis

To explore the genes that could predict the prognosis of cancer patients, a risk factor score was calculated to assess the survival of patients. In brief, we used a univariate Cox regression analysis to evaluate the association between survival time and the expression levels of those genes. A mathematical formula was constructed to predict survival (Li et al., 2016), and the risk score of each patient was calculated as follows:

$$R\,i\,s\,k\,\_\,S\,c\,o\,r\,e=\sum_{i=1}^{n}\beta_{i}*e\,x\,p\,r\,e\,s\,s\,i\,o\,n\,\left(g\,e\,n\,e\,s\right)$$

β*_*i*_* represents the regression coefficient of each gene expression value. All patients in the datasets were thus assigned to high-risk and low-risk groups using the median risk score as the cut-off point. The Kaplan-Meier method was used to estimate the overall survival (OS) time for the two subgroups, and differences in survival time were analyzed using the log-rank test (R package “survival”).

### Statistical Analysis

All statistical analyses in this study were performed with R software. The UpSet plot was generated by the R package “UpSetR.” All statistical tests were two-sided, and *p* < 0.05 was considered significant.

## Results

### Computation of Gene Co-expression Relationships in Villi and Tumors and Functional Enrichment Analyses

For each gene in villi and tumors, we used the Matrix eQTL engine function in the “MatrixEQTL” package to calculate the correlation between each row of the two matrices by linear regression, in other words the associations between pairwise gene expression levels, and tested its significance with t statistics. To enable quantitative comparisons of gene co-expression relationships in villi and cancer, we standardized the number of edges in each network by constructing networks from the most significant 10,000 interactions in villi and tumors.

**FIGURE 1:**
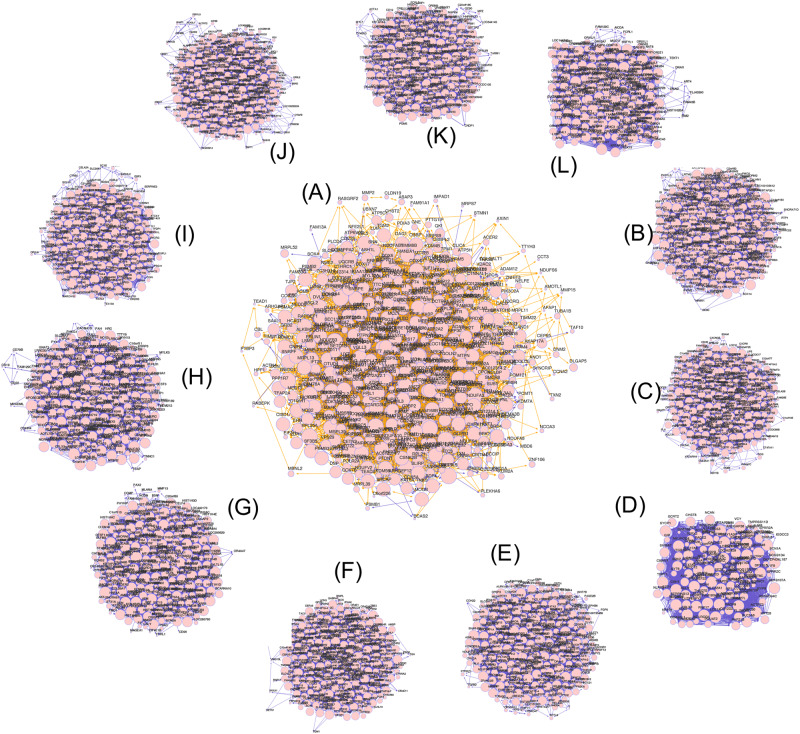
Gene co-expression networks of villi and multiple tumors. **(A)** The gene co-expression networks of villi. **(B–L)** The gene co-expression networks of BLCA, BRCA, COAD, READ, EAC, GBMLGG, HNSC, LUAD, STAD, ESCC, and LUSC, respectively. The edge weight indicates the statistical significance of the gene co-expression relationship, the edge color indicates the direction of the association (positive shown in yellow; negative shown in blue), and the node size indicates the node degree.

To visualize the gene co-expression networks, we used the “RedeR” package and limited the analysis to genes with a network degree greater than ten (Figure 1). To systematically test for network functional enrichment and to directly compare network functional enrichment in villi and tumors, we used the SANTA method to test the association between a query gene set and a network, enabling the functional annotation of networks. By determining the association between hallmark gene sets and networks in villi and cancers, these results demonstrated that the villi and cancer co-expression networks were enriched for EMT gene sets, and the *p-*values of EMT enrichment in villi and 11 cancers are shown in Table 2.

**TABLE 2:** The *p-*value enriched by EMT in villi and 11 cancers.

Tissue type	*p-*value
villi.early	0.00000
BLCA	0.00000
BRCA	0.00000
COAD	0.00272
ECA	0.01548
ESCC	0.00001
GBMLGG	0.00083
HNSC	0.00002
LUAD	0.00000
LUSC	0.26326
READ	0.00000
STAD	0.00000

### Identification of “Off-Track” and Highly Expressed Genes in Cancers

**FIGURE 2:**
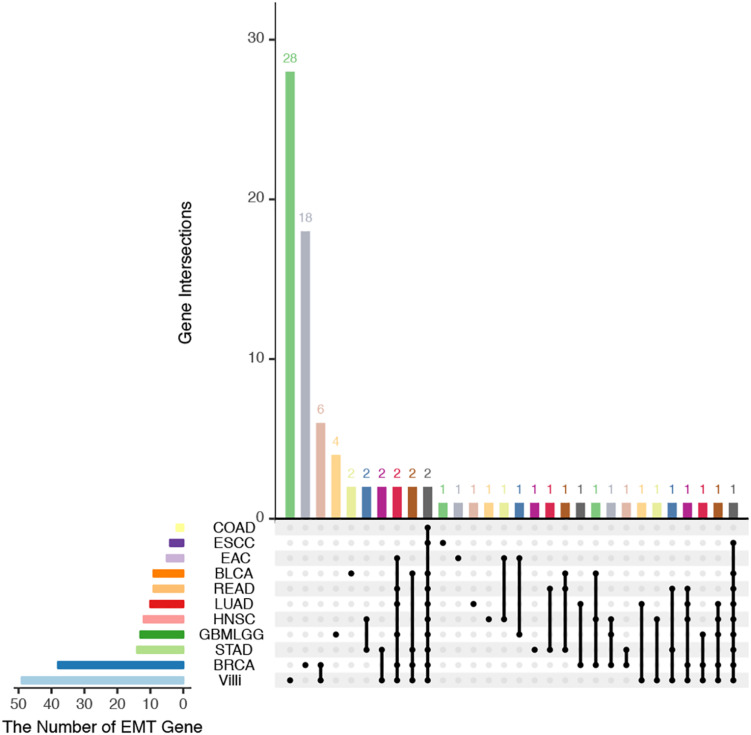
UpSet plot of the intersection of EMT-related genes between villi and cancers. The transverse bar graph at the bottom left shows the number of EMT-related genes in each set. The black points in the dot matrix at the bottom left and in the bar graph at the top indicate genes included in the correlation set. The bars above represent the number of genes corresponding to each intersection.

**FIGURE 3:**
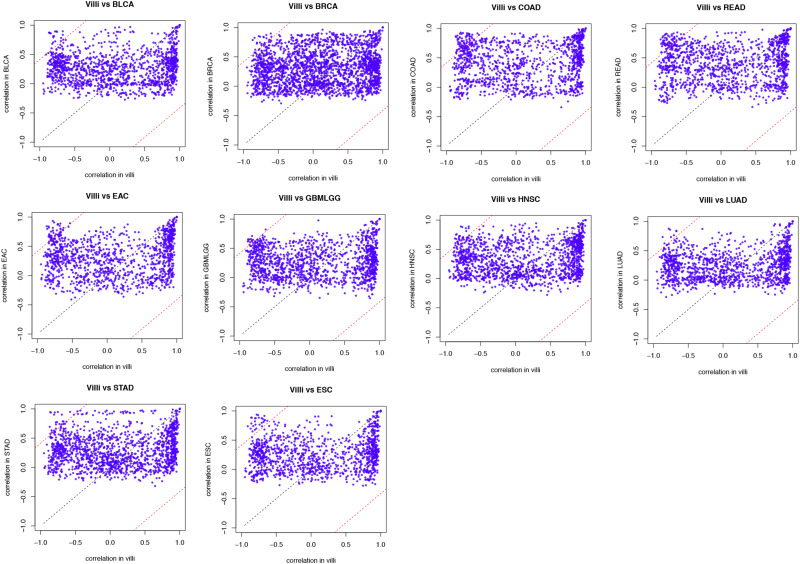
Identified “off-track genes” in villi and tumors. In the biological process of EMT, the interaction between gene pairs in villi and a specific type of cancer was projected on the coordinate axes. The black dotted line in the figure represents the line *y* = *x*, and a distance of 1 from this line is shown by the red dotted line.

The developing placental villi and cancers networks were both enriched in EMT; in other words, they undergo a similar EMT process, but the genes that perform this function are not identical (Figure 2). For example, 49 genes were involved in the EMT in villi development, 38 genes were involved in BRCA, and 16 EMT-related genes were involved in both processes, accounting for 32.7% and 42.1% of the EMT genes involved in villi and BRCA, respectively. Then, according to the “off-track theory,” we identified the “off-track genes” in multiple cancers (Figure 3).

To further screen genes to assess the prognosis of cancer patients, we used Student’s *t*-test to identify differentially expressed genes in cancers. The genes that were significantly upregulated and downregulated in multiple cancers are shown in Table 3.

**TABLE 3:** High and low expression of off-track genes in each tumor.

Tumor	High-expression genes	Low-expression genes
BLCA	MMP14, COL5A2, CTHRC1, VCAN	ACTA2, DCN, FERMT2, FMOD, ITGA5, LUM, MMP2, MYL9, TAGLN, TPM1
BRCA	CDH11, COL12A1, COL1A1, COL1A2, COL3A1, COL5A2, COL8A2, CTHRC1, LUM, MMP14	DCN, ECM2, FERMT2
COAD	CALU, COL1A1, COL1A2, COL3A1, COL4A1, COL4A2, COL5A2, CTHRC1, LUM, MMP14, MMP2, PLOD1, VCAN	FERMT2, TPM1, EMP3, VIM, ACTA2, DCN, IGFBP4, MGP, MYL9, TAGLN, SNAI2, PMP22, FBLN2
READ	CALU, COL1A1, COL1A2, COL3A1, COL4A1, COL5A2, CTHRC1, MMP14, MMP2, VCAN	ACTA2, CALD1, DCN, FBLN2, FERMT2, MGP, MYL9, PMP22, TAGLN, TPM1, VIM
EAC	COL1A1, COL3A1, COL4A1, COL5A2, CTHRC1, MMP14	NA
ESCC	COL1A1, COL1A2, COL3A1, COL5A2, CTHRC1, LUM, MMP14, VCAN	NA
GBMLGG	ACTA2, COL1A1, COL1A2, COL3A1, COL5A2, COL6A2, ITGB1, LUM, MMP14, PLOD1, SNAI2, TAGLN, VIM	NA
HNSC	MMP2, COL1A1, COL1A2, COL3A1, COL5A2, COL6A2, CTHRC1, LUM, MMP14, PCOLCE, VCAN	TPM1
LUAD	COL1A1, COL1A2, COL3A1, COL5A2, LUM, MMP2	ACTA2, DCN
PRAD	CTHRC1	ACTA2, COL4A1, COL4A2, COL6A2, DCN, EMP3, FERMT2, IGFBP4, ITGA5, LUM, MATN2, MMP14, MMP2, MYL9, PMP22, TAGLN, TPM1, VIM
STAD	COL1A2, COL3A1, COL5A2, CTHRC1, LUM, MMP14, MMP2	CALD1, FERMT2, TPM1, ACTA2, TAGLN, SFRP1, MYL9, MYLK, DCN, FLNA

### Prognostic Significance of “Off-Track Genes” in Cancer Patients

**FIGURE 4:**
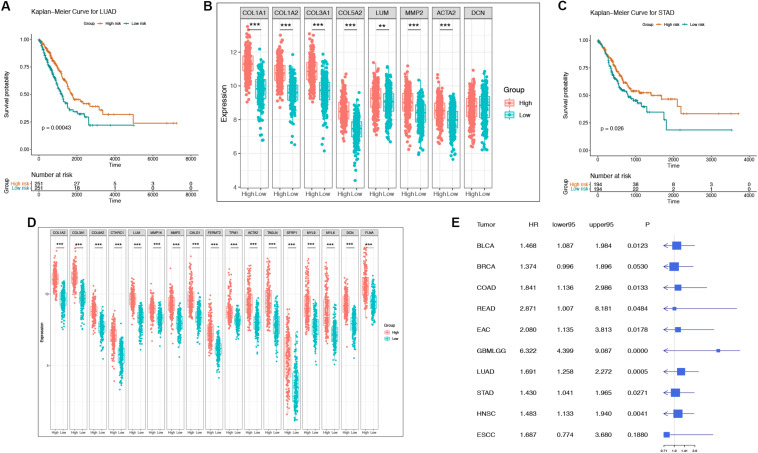
The representative survival curve and forest plot of “off-track gene” risk scores for multiple cancers. **(A–B)** The survival curves and the gene expression intensity in the low risk group as well as in the high risk group of LUAD patients in TCGA datasets. *P < 0.05, **P < 0.01, ***P < 0.001, and no stars for genes whose *p*-value is > 0.05. Box plots compare the difference of gene expression between risk groups using a *t*-test. **(C–D)** The survival curves and the gene expression intensity in the low risk group as well as in the high risk group of STAD patients in TCGA datasets. *P < 0.05, **P < 0.01, ***P < 0.001, and no stars for genes whose *p*-value is > 0.05. Box plots compare the difference of gene expression between risk groups using a *t*-test. **(E)** Forest plot of “off-track genes” using data from multiple cancer cohorts in TCGA using a univariate cox repression model. The forest plot shows the HR, 95% confidence interval, and *p-*value for each type of cancer.

To determine whether these “off-track genes” were related to the prognosis of cancer patients, we used Kaplan-Meier curves for the analysis. A log-rank test confirmed that the risk score of “off-track genes” was significantly related to the OS of cancer patients, and a higher risk score suggested a poor prognosis (BLCA, *n* = 408, *p* = 0.008; BRCA, *n* = 1093, *p* = 0.052; COAD, *n* = 279, *p* = 0.012; EAC, *n* = 89, *p* = 0.016; ESCC, *n* = 95, *p* = 0.184; GBMLGG, *n* = 421, *p* = 1.70e–29; HNSC, *n* = 520, *p* = 0.004; LUAD, *n* = 502, *p* = 4.28e–04; READ, *n* = 93, *p* = 0.039; and STAD, *n* = 388, *p* = 0.026). These survival curves and differences in their gene expression of representative respiratory and digestive tract tumors are shown in Figures 4A–D. To further assess the impact of “off-track genes” on cancer patients’ survival, we performed univariate and multivariate Cox regressions; the results demonstrated that high risk scores for these genes increased the risk of death in cancer patients, and these off-track genes were independent prognostic factors in cancers except BLCA. In addition, high risk scores for these genes in ESCC were not related to patient survival. The results of the Cox proportional hazards regression analysis of clinical characteristics (including age, gender, and tumor stages) are presented in Supplementary Tables S2–S11, and the results of univariate Cox regression of the “off-track gene” risk scores are shown in Figure 4E.

**FIGURE 5:**
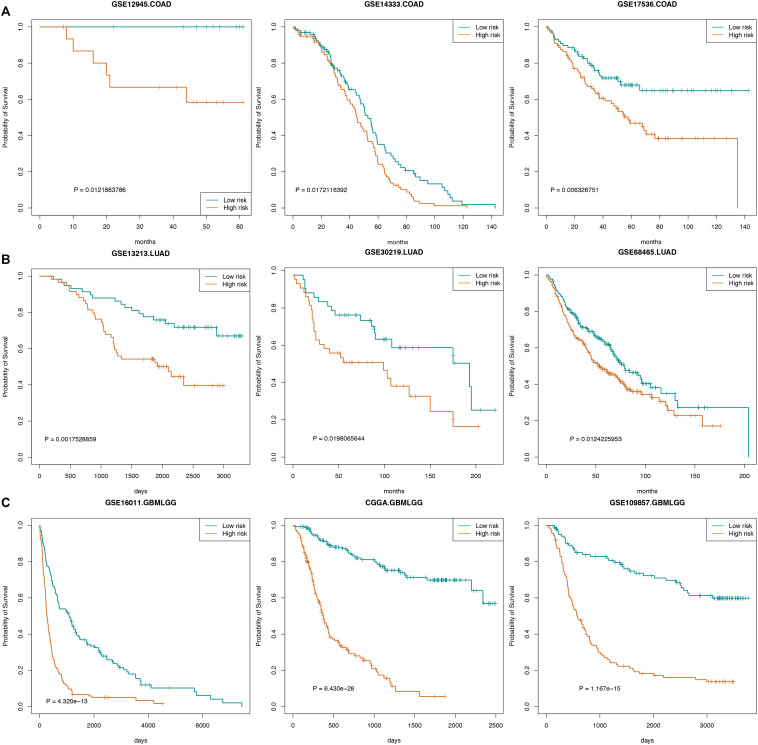
Survival curves for cancers. **(A)** The survival curve of COAD patients in GSE14333, GSE12945, and GSE17536. **(B)** The survival curve of LUAD patients in GSE30219, GSE13213, and GSE68465. **(C)** The survival curve of GBMLGG patients in GSE109857, GSE16011, and CGGA.

We also used certain independent cancer datasets to validate the prognostic value of these “off-track genes.” The survival analysis results revealed that the risk scores of “off-track genes” were significantly correlated with cancer patient survival, such as in the COAD datasets (GSE14333, *n* = 196, *p* = 0.017; GSE12945, *n* = 29, *p* = 0.012; and GSE17536, *n* = 177, *p* = 0.006), LUAD datasets (GSE30219, *n* = 85, *p* = 0.020; GSE13213, *n* = 117, *p* = 0.002; and GSE68465, *n* = 443, *p* = 0.012), and GBMLGG datasets (GSE109857, *n* = 225, *p* = 1.167e-15; GSE16011, *n* = 284, *p* = 4.320e-13; and CGGA, *n* = 325, *p* = 6.430e-28). These survival curves are shown in Figure 5.

## Discussion

Increasing evidences support the atavistic theory of cancer, that is, the biological origin of cancer can be found in the early transitional phase from unicellularity to multicellularity (Bussey et al., 2017; Trigos et al., 2017). Tumorigenesis and placental villi development exhibit many similar biological behaviors, but their outcomes are different. Gene co-expression networks are the basis of embryonic development and tumorigenesis (Singh et al., 2017), and the gene regulatory network in tumors becomes disrupted (Bussey et al., 2017).

The EMT during placental development is a normal physiological process that involves a strong correlation between genes, suggesting that all biological functions are under the tightly precise regulation of gene networks. However, during the process of tumor progression, the close relationship of genes is disrupted and becomes “off-track” from that in normal placental development. Therefore, we proposed the “off-track theory” for the first time. Such “off-track genes” are likely to promote the progression of cancer and may also predict the prognosis of cancer patients.

Different gene co-expression networks lead to different biological outcomes, and this finding has received increased attention from researchers. However, methods to measure changes in the network have not been well explored. In this study, we focused on biological processes involved in the EMT, considering a recent paper “Guidelines and definitions for research on epithelial–mesenchymal transition” (Yang et al., 2020), which authors put forward Consensus Statement about EMT and suggest use cellular properties together with a set of molecular markers to define the EMT status. We used 200 EMT related markers from the MSigDB and identified “off-track genes” in cancer networks using the “off-track theory.” First, we used Pearson’s correlation analysis to calculate the correlation coefficient of pairs of genes that are involved in the EMT in villi and cancer development. The correlation coefficient of genes in biological networks can reflect the degree of biological connection (Teschendorff et al., 2010; Bockmayr et al., 2013). Then, Pearson’s correlation coefficients of the EMT-related gene pairs in villi and cancer were projected on a coordinate axis to identify the gene pairs with a distance from the line *y* = *x* greater than 1. Based on the results, we identified “off-track genes” in multiple cancers and deemed that these genes were associated with tumor progression or the prognosis.

To further evaluate the relationship between “off-track genes” and the prognosis of patients, we performed a survival analysis. A high risk score of these genes was significantly associated with poor prognosis in all forms of adenocarcinoma that we analyzed as well as GBMLGG and HNSC, but it was not associated with the prognosis for LUSC and ESCC. There are several reasons that might explain the different prognostic values. First, endoderm-derived organ tumors are more closely related to embryonic development. The endoderm generates the digestive tube and its accessory organs, the respiratory and pulmonary epithelium, the thymus and the bladder; in addition, nerve cells also originate from the endoderm (Solnica-Krezel, 2005). Second, LUSC has been reported to possibly be derived from basal cells (Sutherland and Berns, 2010; Terry et al., 2010); it exhibits a stepwise progression from normal bronchial epithelium to squamous metaplasia to LUSC, and it has a different development process than LUAD (Nishisaka et al., 2000). Third, ESCC is caused by abnormal hyperplasia of esophageal squamous epithelium, which does not appear during embryonic development (di Pietro et al., 2018). Fourth, HNSC was the only type of squamous cell carcinoma associated with a significantly poor prognosis in this study; however, stage IV patients accounted for 59% of the total HNSC patients, which may influence the survival results.

The results of this study requires further verification. First, although we confirmed that the co-expression network is altered in the cancerous process compared with normal physiological processes and identified the “off-track genes,” the network relationships are very complex, and it is difficult to determine a standard for measuring the changes in network relationships. Further exploration is urgently needed. Second, these identified off-track EMT related genes not validated in tumors *in vitro* and *in vivo*. This requires a lot of work, and it is hard to verify every gene in every cancer, further studies are planned. Third, the transcriptome represents only one level of the biological process and cannot fully reflect the function of the entire biological system. These results should be combined with other “omics” data, such as proteomics and methylation data, to conduct an integrated analysis of multi-omics data to provide more comprehensive information on the biological system.

The placental villi development model may be a useful tool for exploring tumorigenesis. Development models are widely used to study the complex mechanisms of tumorigenesis, and many important signaling pathways and molecular markers have been found (Kho et al., 2004; Liu et al., 2006; Kaiser et al., 2007).

Our study indicate that villus development is a reliable and strictly regulated model that can illuminate the trajectory of human cancer development, and gene co-expression in disease states is “off-track” from that in the normal state. The “off-track genes” may have a substantial impact on tumor development and reveal novel prognostic biomarkers to assist in cancer treatment and evaluate the therapeutic effect.

## Data Availability Statement

The datasets generated for this study can be found in the Genome Sequence Archive in the Beijing Institute of Genomics (BIG) Data Center, BIG, Chinese Academy of Sciences, under the accession number HRA000050.

## Ethics Statement

The studies involving human participants were reviewed and approved by Cancer Institute and Hospital, Chinese Academy of Medical Sciences. The patients/participants provided their written informed consent to participate in this study. Written informed consent was obtained from the individual(s) for the publication of any potentially identifiable images or data included in this article.

## Author Contributions

SC and WL conceived and designed the study. BZ extracted RNA, performed the data analysis, and wrote the manuscript. HL and YW collected villi tissues. LF provided advice on the study. All authors contributed to the article and approved the submitted version.

## Conflict of Interest

The authors declare that the research was conducted in the absence of any commercial or financial relationships that could be construed as a potential conflict of interest.

## Footnotes

1 https://en.novogene.com/

## Supplementary Material

TABLE S1 The detailed information of villi samples.

TABLE S2 Cox proportional hazards regression analysis of OS in BLCA.

TABLE S3 Cox proportional hazards regression analysis of OS in BRCA.

TABLE S4 Cox pro portional hazards regression analysis of OS in COAD.

TABLE S5 Cox proportional hazards regression analysis of OS in EAC.

TABLE S6 Cox proportional hazards regression analysis of OS in ESCC.

TABLE S7 Cox proportional hazards regression analysis of OS in GBMLGG.

TABLE S8 Cox proportional hazards regression analysis of OS in HNSC.

TABLE S9 Cox proportional hazards regression analysis of OS in LUAD.

TABLE S10 Cox proportional hazards regression analysis of OS in READ.

TABLE S11 Cox proportional hazards regression analysis of OS in STAD.
