# Supplementary material for: Identification of Prognostic Biomarkers for Multiple Solid Tumors Using a Human Villi Development Model
Source: Front Cell Dev Biol. 2020 Jun 23;8:492. doi: 10.3389/fcell.2020.00492 (PMC7325693; doi:10.3389/fcell.2020.00492)
Supplement: TABLE S2 — Cox proportional hazards regression analysis of OS in BLCA. [file Table_2.DOCX]

Table S2. Cox proportional hazards regression analysis of OS in BLCA

| Parameters | **Univariate cox regression** | | | | |  | **Multivariate cox regression** | | |
| --- | --- | --- | --- | --- | --- | --- | --- | --- | --- |
|  | HR | | 95% CI | | *P* |  | HR | 95% CI | *P* |
| Age | | 1.033 | | 1.018-1.049 | **0.000** |  | 1.029 | 1.013-1.045 | **0.000** |
| Gender (M/F) ^a^ | | 0.879 | | 0.635-1.216 | 0.435 |  | 0.866 | 0.624-1.202 | 0.390 |
| Stage | |  | |  |  |  |  |  |  |
| III vs I | | 1.581 | | 1.037-2.412 | **0.033** |  | 1.461 | 0.947-2.253 | 0.086 |
| IV vs I | | 2.857 | | 1.926-4.237 | **0.000** |  | 2.593 | 1.730-3.886 | **0.000** |
| Genes (H vs L) ^b^ | | 1.468 | | 1.087-1.984 | **0.012** |  | 1.220 | 0.895-1.665 | 0.208 |

HR, Hazard ration; 95% CI, 95% confidence interval.

^a^ M: Male, F: Female.

^b^ H: High risk scores, L: Low risk scores.
