# Supplementary material for: Identification of Prognostic Biomarkers for Multiple Solid Tumors Using a Human Villi Development Model
Source: Front Cell Dev Biol. 2020 Jun 23;8:492. doi: 10.3389/fcell.2020.00492 (PMC7325693; doi:10.3389/fcell.2020.00492)
Supplement: TABLE S3 — Cox proportional hazards regression analysis of OS in BRCA. [file Table_3.DOCX]

Table S3. Cox proportional hazards regression analysis of OS in BRCA

| Parameters | **Univariate cox regression** | | | | |  | **Multivariate cox regression** | | |
| --- | --- | --- | --- | --- | --- | --- | --- | --- | --- |
|  | HR | | 95% CI | | *P* |  | HR | 95% CI | *P* |
| Age | | 1.032 | | 1.019-1.045 | **0.000** |  | 1.035 | 1.022-1.048 | **0.000** |
| Gender (M/F) ^a^ | | 0.839 | | 0.117-6.012 | 0.862 |  | 0.550 | 0.076-3.964 | 0.552 |
| Stage | |  | |  |  |  |  |  |  |
| II vs I | | 1.597 | | 0.925-2.759 | 0.093 |  | 1.871 | 1.079-3.245 | **0.026** |
| III vs I | | 3.065 | | 1.730-5.428 | **0.000** |  | 3.721 | 2.088-6.631 | **0.000** |
| IV vs I | | 12.951 | | 6.385-26.266 | **0.000** |  | 11.185 | 5.502-22.735 | **0.000** |
| CHPF (H vs L) ^b^ | | 1.374 | | 0.995-1.896 | 0.053 |  | 1.417 | 1.019-1.971 | **0.038** |

HR, Hazard ration; 95% CI, 95% confidence interval.

^a^ M: Male, F: Female.

^b^ H: High High risk scores, L: Low risk scores.
