# Supplementary material for: Identification of Prognostic Biomarkers for Multiple Solid Tumors Using a Human Villi Development Model
Source: Front Cell Dev Biol. 2020 Jun 23;8:492. doi: 10.3389/fcell.2020.00492 (PMC7325693; doi:10.3389/fcell.2020.00492)
Supplement: TABLE S4 — Cox pro portional hazards regression analysis of OS in COAD. [file Table_4.DOCX]

Table S4. Cox proportional hazards regression analysis of OS in COAD

| Parameters | **Univariate cox regression** | | | | |  | **Multivariate cox regression** | | |
| --- | --- | --- | --- | --- | --- | --- | --- | --- | --- |
|  | HR | | 95% CI | | *P* |  | HR | 95% CI | *P* |
| Age | | 1.015 | | 0.996-1.034 | 0.128 |  | 1.031 | 1.009-1.054 | **0.005** |
| Gender (M/F) ^a^ | | 1.486 | | 0.912-2.423 | 0.113 |  | 1.261 | 0.762-2.085 | 0.367 |
| Stage | |  | |  |  |  |  |  |  |
| II vs I | | 2.327 | | 0.694-7.805 | **0.034** |  | 2.381 | 0.708-8.009 | 0.161 |
| III vs I | | 3.712 | | 1.106-12.465 | **0.000** |  | 4.322 | 1.277-14.628 | **0.019** |
| IV vs I | | 9.878 | | 2.918-33.444 | **0.005** |  | 12.959 | 3.706-45.318 | **0.000** |
| CHPF (H vs L) ^b^ | | 1.841 | | 1.136-2.986 | **0.013** |  | 1.642 | 0.999-2.697 | **0.000** |

HR, Hazard ration; 95% CI, 95% confidence interval.

^a^ M: Male, F: Female.

^b^ H: High High risk scores, L: Low risk scores.
