# Supplementary material for: Identification of Prognostic Biomarkers for Multiple Solid Tumors Using a Human Villi Development Model
Source: Front Cell Dev Biol. 2020 Jun 23;8:492. doi: 10.3389/fcell.2020.00492 (PMC7325693; doi:10.3389/fcell.2020.00492)
Supplement: TABLE S5 — Cox proportional hazards regression analysis of OS in EAC. [file Table_5.DOCX]

Table S5. Cox proportional hazards regression analysis of OS in EAC

| Parameters | **Univariate cox regression** | | | | |  | **Multivariate cox regression** | | |
| --- | --- | --- | --- | --- | --- | --- | --- | --- | --- |
|  | HR | | 95% CI | | *P* |  | HR | 95% CI | *P* |
| Age | | 0.982 | | 0.958-1.006 | 0.141 |  | 0.986 | 0.960-1.013 | 0.300 |
| Gender (M/F) ^a^ | | 1.064 | | 0.377-3.007 | 0.907 |  | 0.470 | 0.155-1.426 | 0.182 |
| Stage | |  | |  |  |  |  |  |  |
| II vs I | | 2.964 | | 0.832-10.56 | 0.094 |  | 4.986 | 1.311-18.957 | **0.018** |
| III vs I | | 3.047 | | 0.857-10.84 | 0.085 |  | 5.162 | 1.319-20.197 | **0.018** |
| IV vs I | | 5.912 | | 1.369-25.53 | **0.017** |  | 6.100 | 1.288-28.898 | **0.023** |
| CHPF (H vs L) ^b^ | | 2.080 | | 1.135-3.813 | **0.018** |  | 2.778 | 1.419-5.439 | **0.003** |

HR, Hazard ration; 95% CI, 95% confidence interval.

^a^ M: Male, F: Female.

^b^ H: High High risk scores, L: Low risk scores.
