# Supplementary material for: Identification of Prognostic Biomarkers for Multiple Solid Tumors Using a Human Villi Development Model
Source: Front Cell Dev Biol. 2020 Jun 23;8:492. doi: 10.3389/fcell.2020.00492 (PMC7325693; doi:10.3389/fcell.2020.00492)
Supplement: TABLE S6 — Cox proportional hazards regression analysis of OS in ESCC. [file Table_6.DOCX]

Table S6. Cox proportional hazards regression analysis of OS in ESCC

| Parameters | **Univariate cox regression** | | | | |  | **Multivariate cox regression** | | |
| --- | --- | --- | --- | --- | --- | --- | --- | --- | --- |
|  | HR | | 95% CI | | *P* |  | HR | 95% CI | *P* |
| Age | | 1.001 | | 0.964-1.039 | 0.969 |  | 0.993 | 0.954-1.035 | 0.752 |
| Gender (M/F) ^a^ | | 1.861 | | 0.441-7.849 | 0.398 |  | 1.940 | 0.398-9.454 | 0.412 |
| Stage | |  | |  |  |  |  |  |  |
| II vs I | | 0.826 | | 0.185-3.69 | 0.802 |  | 0.614 | 0.120-3.135 | 0.558 |
| III vs I | | 1.315 | | 0.289-5.986 | 0.724 |  | 0.992 | 0.190-5.194 | 0.993 |
| IV vs I | | 1.601 | | 0.254-10.109 | 0.617 |  | 1.225 | 0.174-8.627 | 0.839 |
| CHPF (H vs L) ^b^ | | 1.687 | | 0.774-3.68 | 0.188 |  | 1.650 | 0.734-3.710 | 0.225 |

HR, Hazard ration; 95% CI, 95% confidence interval.

^a^ M: Male, F: Female.

^b^ H: High High risk scores, L: Low risk scores.
