# Supplementary material for: Identification of Prognostic Biomarkers for Multiple Solid Tumors Using a Human Villi Development Model
Source: Front Cell Dev Biol. 2020 Jun 23;8:492. doi: 10.3389/fcell.2020.00492 (PMC7325693; doi:10.3389/fcell.2020.00492)
Supplement: TABLE S7 — Cox proportional hazards regression analysis of OS in GBMLGG. [file Table_7.DOCX]

Table S7. Cox proportional hazards regression analysis of OS in GBMLGG

| Parameters | **Univariate cox regression** | | | | |  | **Multivariate cox regression** | | |
| --- | --- | --- | --- | --- | --- | --- | --- | --- | --- |
|  | HR | | 95% CI | | *P* |  | HR | 95% CI | *P* |
| Age | | 1.066 | | 1.054-1.079 | **<2e-16** |  | 1.039 | 1.025-1.052 | **1.08E-08** |
| Gender (M/F) ^a^ | | 1.326 | | 0.984-1.787 | 0.060 |  | 0.911 | 0.670-1.239 | 0.554 |
| WHO | |  | |  |  |  |  |  |  |
| IV vs II | | 8.194 | | 5.933-11.32 | **<2e-16** |  | 2.656 | 1.785-3.952 | **1.45E-06** |
| CHPF (H vs L) ^b^ | | 6.322 | | 4.399-9.087 | **<2e-16** |  | 3.108 | 2.011-4.805 | **3.33E-07** |

HR, Hazard ration; 95% CI, 95% confidence interval.

^a^ M: Male, F: Female.

^b^ H: High High risk scores, L: Low risk scores.
