# Supplementary material for: Identification of Prognostic Biomarkers for Multiple Solid Tumors Using a Human Villi Development Model
Source: Front Cell Dev Biol. 2020 Jun 23;8:492. doi: 10.3389/fcell.2020.00492 (PMC7325693; doi:10.3389/fcell.2020.00492)
Supplement: TABLE S8 — Cox proportional hazards regression analysis of OS in HNSC. [file Table_8.DOCX]

Table S8. Cox proportional hazards regression analysis of OS in HNSC

| Parameters | **Univariate cox regression** | | | | |  | **Multivariate cox regression** | | |
| --- | --- | --- | --- | --- | --- | --- | --- | --- | --- |
|  | HR | | 95% CI | | *P* |  | HR | 95% CI | *P* |
| Age | | 1.017 | | 1.006-1.029 | **0.004** |  | 1.019 | 1.006-1.031 | **0.004** |
| Gender (M/F) ^a^ | | 0.745 | | 0.559-0.9919 | **0.044** |  | 0.842 | 0.621-1.142 | 0.269 |
| Stage | |  | |  |  |  |  |  |  |
| II vs I | | 3.599 | | 1.263-10.254 | **0.017** |  | 3.653 | 1.282-10.409 | **0.015** |
| III vs I | | 2.485 | | 0.873-7.075 | 0.088 |  | 2.644 | 0.926-7.548 | 0.069 |
| IV vs I | | 4.260 | | 1.574-11.53 | **0.004** |  | 4.555 | 1.681-12.346 | **0.003** |
| CHPF (H vs L) ^b^ | | 1.483 | | 1.133-1.940 | **0.004** |  | 1.478 | 1.119-1.952 | **0.006** |

HR, Hazard ration; 95% CI, 95% confidence interval.

^a^ M: Male, F: Female.

^b^ H: High High risk scores, L: Low risk scores.
