# Supplementary material for: Identification of Prognostic Biomarkers for Multiple Solid Tumors Using a Human Villi Development Model
Source: Front Cell Dev Biol. 2020 Jun 23;8:492. doi: 10.3389/fcell.2020.00492 (PMC7325693; doi:10.3389/fcell.2020.00492)
Supplement: TABLE S9 — Cox proportional hazards regression analysis of OS in LUAD. [file Table_9.DOCX]

Table S9. Cox proportional hazards regression analysis of OS in LUAD

| Parameters | **Univariate cox regression** | | | | |  | **Multivariate cox regression** | | | |
| --- | --- | --- | --- | --- | --- | --- | --- | --- | --- | --- |
|  | HR | | 95% CI | | *P* |  | HR | 95% CI | | *P* |
| Age | | 1.007 | | 0.991-1.023 | 0.373 |  | 1.012 | 0.996-1.028 | 0.149 | |
| Gender (M/F) ^a^ | | 1.053 | | 0.786-1.409 | 0.731 |  | 0.987 | 0.727-1.341 | 0.934 | |
| Stage | |  | |  |  |  |  |  |  | |
| II vs I | | 2.426 | | 1.686-3.490 | **1.78E-06** |  | 2.118 | 1.445-3.104 | **1.20E-04** | |
| III vs I | | 3.509 | | 2.393-5.146 | **1.30E-10** |  | 2.900 | 1.953-4.305 | **1.28E-07** | |
| IV vs I | | 3.832 | | 2.209-6.650 | **1.77E-06** |  | 3.835 | 2.184-6.735 | **2.89E-06** | |
| CHPF (H vs L) ^b^ | | 1.691 | | 1.258-2.272 | **4.94E-04** |  | 1.447 | 1.056-1.984 | **0.022** | |

HR, Hazard ration; 95% CI, 95% confidence interval.

^a^ M: Male, F: Female.

^b^ H: High High risk scores, L: Low risk scores.
