# Supplementary material for: Identification of Prognostic Biomarkers for Multiple Solid Tumors Using a Human Villi Development Model
Source: Front Cell Dev Biol. 2020 Jun 23;8:492. doi: 10.3389/fcell.2020.00492 (PMC7325693; doi:10.3389/fcell.2020.00492)
Supplement: TABLE S10 — Cox proportional hazards regression analysis of OS in READ. [file Table_10.DOCX]

Table S10. Cox proportional hazards regression analysis of OS in READ

| Parameters | **Univariate cox regression** | | | | |  | **Multivariate cox regression** | | | |
| --- | --- | --- | --- | --- | --- | --- | --- | --- | --- | --- |
|  | HR | | 95% CI | | *P* |  | HR | 95% CI | | *P* |
| Age | | 1.107 | | 1.048-1.17 | **2.72E-04** |  | 0.674 | 0.192-2.365 | 0.538 | |
| Gender (M/F) ^a^ | | 0.621 | | 0.233-1.655 | 0.341 |  | 1.108 | 1.033-1.189 | **0.004** | |
| Stage | |  | |  |  |  |  |  |  | |
| II vs I | | 0.222 | | 0.020-2.487 | 0.222 |  | 0.110 | 0.009-1.33 | 0.083 | |
| III vs I | | 1.692 | | 0.350-8.186 | 0.513 |  | 0.503 | 0.084-3.013 | 0.451 | |
| IV vs I | | 2.028 | | 0.333-12.331 | 0.443 |  | 0.699 | 0.093-5.251 | 0.728 | |
| CHPF (H vs L) ^b^ | | 2.871 | | 1.007-8.181 | **0.048** |  | 3.831 | 1.184-12.393 | **0.025** | |

HR, Hazard ration; 95% CI, 95% confidence interval.

^a^ M: Male, F: Female.

^b^ H: High High risk scores, L: Low risk scores.
