# Supplementary material for: Identification of Prognostic Biomarkers for Multiple Solid Tumors Using a Human Villi Development Model
Source: Front Cell Dev Biol. 2020 Jun 23;8:492. doi: 10.3389/fcell.2020.00492 (PMC7325693; doi:10.3389/fcell.2020.00492)
Supplement: TABLE S11 — Cox proportional hazards regression analysis of OS in STAD. [file Table_11.DOCX]

Table S11. Cox proportional hazards regression analysis of OS in STAD

| Parameters | **Univariate cox regression** | | | | |  | **Multivariate cox regression** | | | |
| --- | --- | --- | --- | --- | --- | --- | --- | --- | --- | --- |
|  | HR | | 95% CI | | *P* |  | HR | 95% CI | | *P* |
| Age | | 1.020 | | 1.005-1.035 | **0.011** |  | 1.029 | 1.013-1.045 | 4.33E-04 | |
| Gender (M/F) ^a^ | | 1.228 | | 0.876-1.722 | 0.234 |  | 1.166 | 0.829-1.641 | 0.379 | |
| Stage | |  | |  |  |  |  |  |  | |
| II vs I | | 1.542 | | 0.801-2.971 | 0.195 |  | 1.570 | 0.812-3.033 | 0.180 | |
| III vs I | | 2.538 | | 1.380-4.668 | **0.003** |  | 2.603 | 1.409-4.809 | **0.002** | |
| IV vs I | | 3.908 | | 1.943-7.860 | **0.001** |  | 5.181 | 2.529-10.612 | **6.92E-06** | |
| CHPF (H vs L) ^b^ | | 1.430 | | 1.041-1.965 | **0.027** |  | 1.446 | 1.041-2.007 | **0.028** | |

HR, Hazard ration; 95% CI, 95% confidence interval.

^a^ M: Male, F: Female.

^b^ H: High High risk scores, L: Low risk scores.
